# Background and evaluation design of a community-based health-promoting school intervention: Fit Lifestyle at School and at Home (FLASH)

**DOI:** 10.1186/s12889-019-7088-3

**Published:** 2019-06-20

**Authors:** Bonnie Maria van Dongen, Monica Antonia Maria Ridder, Ingrid Hendrika Margaretha Steenhuis, Carry Mira Renders

**Affiliations:** 1 Department of Health Sciences, Faculty of Science, Vrije Universiteit Amsterdam, Amsterdam Public Health Research Institute, Amsterdam, the Netherlands; 2 Human Movement and Education Division, Windesheim University of Applied Sciences, Zwolle, the Netherlands; 3 Research Center Healthy Cities, Knowledge Center for Health and Social work, Windesheim University of Applied Sciences, Zwolle, the Netherlands

**Keywords:** Community-based approach, Adolescents, Physical activity, Dietary behaviour, Implementation, Mixed methods, Community capacity, Health-promoting school, Pre-vocational secondary school

## Abstract

**Background:**

A community-based approach can be a promising strategy for implementing school-based health promotion aimed at stimulating healthy physical activity and dietary behaviour. Such an approach builds on the community capacity of multiple stakeholders, empowering them to design and implement tailored activities, supported by the whole school community. This paper describes the background and evaluation design of the community-based school intervention ‘Fit Lifestyle at School and at Home’ (FLASH) in four prevocational schools. FLASH includes four strategies for building the community capacity of students, school personnel and parents: 1) identifying leaders in each stakeholder group, 2) stimulating a school culture of participation, 3) having stakeholders design and implement tailored activities and 4) creating a network of local partners for structural embedding. The objective is to monitor the capacity-building processes of the FLASH intervention and to explore if these processes contribute to changes in community capacity. In addition, we will explore if the FLASH intervention is related to changes in PA, dietary behaviours and BMI of students.

**Methods:**

This study has a mixed methods design and uses a participatory action-oriented approach to monitor and evaluate changes in community capacity, tailored health-promotion activities and implementation processes. Methods include semi-structured interviews, focus groups, journals, document analysis and observational scans of the physical environment. In addition, changes in BMI, physical activity and dietary behaviours of prevocational students will be explored by comparing the four intervention schools to four control schools. Data are collected by questionnaires and anthropometric measurements.

**Discussion:**

The main strength of this study is its use of mixed methods to evaluate real-life processes of creating a healthy-school community. This will provide valuable information on capacity-building strategies for the structural embedding of health-promotion activities within school settings. The results could help schools become more empowered to adapt and adopt integral health-promotion interventions in daily practice that suit the needs of their communities, that are expected to be sustainable and that could lead to favourable changes in the PA and dietary behaviour of students.

**Trial registration:**

ISRCTN67201841; date registered: 09-05-2019, retrospectively registered.

**Electronic supplementary material:**

The online version of this article (10.1186/s12889-019-7088-3) contains supplementary material, which is available to authorized users.

## Background

Adolescents are an important target group for overweight prevention. As they grow older, they become more independent and autonomous in making decisions on health behaviour, and they are likely to adopt lifestyle behaviours that are less healthy [1, 2]. In addition, health behaviour developed during this period often persists into adulthood [3], thereby possibly affecting health later in life [4, 5]. School-based health promotion can play an important role in addressing the public-health problem of overweight among adolescents [6, 7].

Many initiatives around the world support health-promoting schools, and schools are increasingly implementing structured, systematic plans to encourage healthy behaviour among their students [8–12]. The World Health Organization and the Schools for Health in Europe network advocate a whole-school approach to encouraging healthy lifestyles within the school setting [13, 14]. The whole-school approach is divided into six components that contribute to integral health promotion within this setting: healthy school policies, physical school environment, social school environment, curriculum with opportunities for developing individual health skills and action competencies, community links, and health services. In the Netherlands, the whole-school approach has been translated into the Dutch Healthy School approach [15]. Schools can earn Healthy School certification for eight health themes if their plans cover the four pillars of an integral approach: 1) health education, 2) health policy, 3) a social and physical environment that stimulates healthy behaviour and 4) a monitoring and referral system for the early identification of health problems or risk behaviour in students [16, 17]. Two themes relate to the prevention of overweight: Physical Activity and Nutrition.

Although several Dutch evidence-based interventions for promoting healthy PA or dietary behaviour are available, the sustainable implementation of these interventions in everyday practice is difficult for schools [18, 19]. This can be an important factor limiting the long-term effects of interventions on the health behaviour and BMI of students in real-life settings [6, 7, 18]. The sustainable implementation of health-promotion activities according to a whole-school approach is limited, because of difficulties associated with a) creating ownership among stakeholders to carry out activities independently and b) tailoring intervention strategies to different school populations and contexts in real-life settings [20–22].

Ownership among stakeholders is limited, as schools have trouble obtaining support from and actively engaging parents and school personnel [16, 23, 24]. They experience difficulties with parental involvement in general with regard to health-promotion topics [16, 25]. Schools and parents may also differ in their perceptions of the role and responsibilities of the school in stimulating healthy behaviours among adolescents [26, 27]. Both parents and teachers experience low self-efficacy in stimulating healthy behaviour in adolescents [27, 28]. Although most teachers consider health promotion important, only a few teachers in a school are actively engaged [29, 30]. A possible explanation is that teachers and other school personnel do not know what role they can play and task they can have or how they can incorporate health issues regarding a healthy lifestyle in their curriculum [31]. They could therefore have limited autonomous motivation to implement interventions and the overarching Dutch Healthy School Approach.

Schools might also feel incapable of tailoring intervention strategies to the needs of their populations and contexts without external support [17, 25]. Evidence-based interventions offer only limited possibilities for adjustments to contextual factors including sociodemographic characteristics of the population, physical environment, resources and school health policies [24, 32]. Furthermore, the limited insight of school personnel into the needs and wants of their students, or into the underlying principles of health-promoting strategies makes it difficult to adjust standard interventions to the factors that motivate and engage students [2, 33].

One promising strategy for designing and implementing school-based health-promotion interventions tailored to the context of the school and the needs of its stakeholders is a community-based approach [34–36]. Such an approach has been shown to increase the involvement of a community that extends beyond the school, and they have even had positive effects on obesity rates in Australia [37]. One characteristic of a community approach is the involvement of multiple stakeholders, who have ownership in designing, implementing and evaluating activities [24, 35]. To co-create health-promotion activities, the group of stakeholders needs community capacity, which entails the capability, motivation and opportunity to identify, prioritize, plan, implement, evaluate and sustain health-promotion activities [38, 39]. The identification of working strategies for capacity-building could potentially contribute to empowering school communities to adapt evidence-based interventions to their own real-life situations. Focusing on building community capacity could be especially useful in the educational system of the Netherlands, which is characterized by decision-making autonomy for schools [40].

For this study, we developed a community-based health-promoting school intervention entitled Fit Lifestyle at School and at Home (FLASH), in collaboration with a local public-health organization and a local educational organization. The educational organization consists of several schools that provide secondary education and vocational education and training. This intervention empowers school communities to design and implement integral school-based health-promotion activities that fit within the four pillars on which the Dutch Healthy School Approach is based and that are intended to stimulate healthy dietary and PA behaviours among students. We selected four pre-vocational schools for the intervention, given that pre-vocational schools are attended by a relatively large group of students at-risk for unhealthy PA and dietary behaviours due to their cultural or socio-economic background [41, 42]. The schools are participating in a three-year intervention aimed at building community capacity in three phases: 1) identifying the needs of stakeholders and opportunities in the school community, 2) creating and carrying out action plans, 3) finding a structural place for and consequently embedding successful activities within the school community.

The objective of the study is to monitor the capacity-building processes of the FLASH intervention, including the design and implementation of health-promotion activities by each school community, and to explore if these processes contribute to changes in community capacity. In addition, we will explore if the FLASH intervention relates to changes is PA, dietary behaviours (including determinants of PA and dietary behaviours) and BMI of students. This paper provides a description of the study design.

## Methods/design

### Conceptual framework

**Fig. 1:**
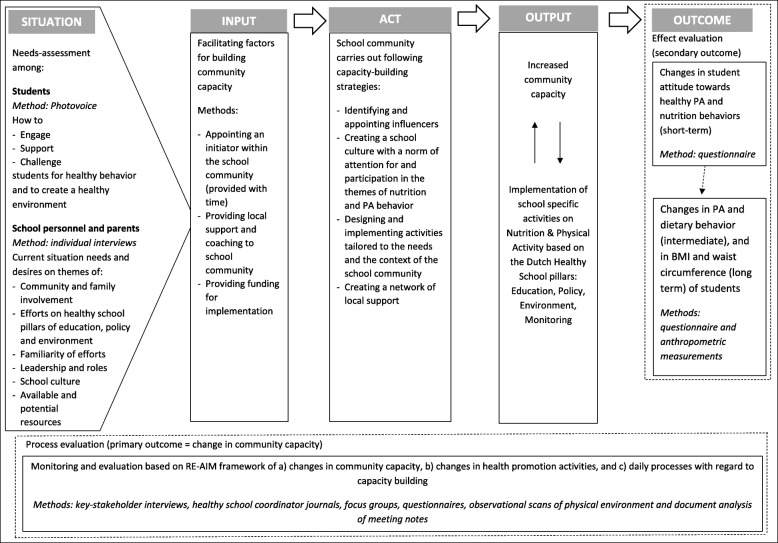
logical model of the FLASH intervention and evaluation study

The logical model displayed in Fig. 1 illustrates how the FLASH intervention creates a healthy-school community and how evaluation data are gathered systematically. The intervention builds on an integral approach based on the whole-school components defined by the WHO and the SHE network, as well as on the Dutch equivalents of these components: the Healthy School pillars. In the FLASH intervention, stakeholders in the school community are defined according to these components: students, school personnel and parents.

The study starts by mapping the current situation of the school community, including the context of the school, the needs, wants and opportunities of all stakeholders involved, and the current PA and dietary behaviours of students. Second, investments are made in realizing several preconditions for facilitating the process of community-capacity building (shown as inputs in Fig. 1) [20, 37].

Third, the school communities engage in capacity-building activities based on four capacity-building strategies [43, 44]: 1) identifying influencers and potential leaders; 2) creating a school culture with attention to PA, dietary behaviour and participation in activities; 3) developing and implementing health-promotion activities that fit into the Healthy School pillars based on the needs of stakeholders and the context of the school; and 4) creating a local network that can support the school community in the long term.

Fourth, the activities generate outputs including increased community capacity and the implementation of tailored health-promotion activities, the ownership of which is held by the school community itself. Finally, the defined output may lead to changes in outcomes in the short, intermediate and long term. Short-term outcomes are defined as changes in student attitudes towards PA and dietary behaviour, with intermediate outcomes defined as changes in the PA and dietary behaviours of students, and long-term outcomes defined as changes in the BMI and waist circumference of students.

### Evaluation design

The study is based on a mixed methods design and uses a participatory action-oriented approach to monitor and evaluate capacity-building processes within the FLASH intervention in four intervention schools. All participating schools are secondary schools providing pre-vocational education. The following goals related to the capacity-building process are monitored and evaluated based on RE-AIM framework elements [45]: a) changes in community capacity, b) changes in health-promotion activities and c) daily processes related to capacity-building throughout the intervention (see Fig. 1). An initial indication of the effects of applying a community-based health-promoting intervention on PA and dietary behaviour of students is explored with a quasi-experimental design by comparing the four intervention schools to four secondary pre-vocational control schools. The intervention’s effects on the health of students are evaluated using a self-reported questionnaire on behaviour and attitudes relating to PA and dietary behaviour, combined with anthropometric measurements. The FLASH intervention and evaluation study were launched in April 2016. The intervention will continue until July 2019, and the evaluation will continue until March 2020. The study protocol has been approved by the Medical Ethics Committee of the VU Medical Centre (reference number 2016.352).

### Intervention

#### Situation: needs-assessment

The FLASH intervention starts with a needs-assessment among a) students and b) key stakeholders (see Fig. 1). For students, the needs-assessment is based on *Photovoice* methodology, which aims to provide a voice to populations who may have trouble expressing themselves (e.g. pre-vocational students) [46]. The methodology also offers the potential to empower this population to assume ownership and take action [47]. Second-year students (12–14 years of age) are asked to make photographs that show what engages, supports and challenges them in making choices regarding PA and dietary behaviour in daily life, especially at school. A self-reported questionnaire also maps the PA and dietary behaviours of students and their attitudes towards these behaviours.

Key stakeholders among the school personnel and parents are interviewed to identify conditions and strengths in the community and opportunities for community-capacity building within their specific contexts. Interview topics are based on the four capacity-building strategies. These interviews also assess the readiness and willingness of each community to create a healthy-school community based on healthy PA and dietary behaviour [44]. The results could help communities to adjust their capacity-building strategies.

#### Input and act: facilitating community-capacity building

The four capacity-building strategies in FLASH are facilitated by the inputs shown in Fig. 1. Table 1 illustrates how each strategy is facilitated. The first strategy (identifying influencers) is facilitated by appointing a healthy-school coordinator, who is allocated time to serve as a leader for the healthy-school community. This coordinator is responsible for creating a network of people representing the community and help to prioritize and design health-promotion activities. By virtue of their teaching or management positions, coordinators are already part of their school communities at the start of the intervention. Existing formal leaders within school communities (e.g. school directors or team managers) are also encouraged to adopt a role in supporting the leaders in the creation of a healthy-school community.

**Table 1:** Overview of capacity-building strategies in FLASH and how these are facilitated

	Capacity-building strategies	Facilitated in FLASH by
1	Identifying and appointing influencers or leaders	- appointing a healthy-school coordinator who knows the community and allocating time to this coordinator for creating a network of motivated initiators - providing coaching and guidance to the coordinator through experts from local organisations
2	Creating a school culture of participation	- the healthy-school coordinator as a starting point - coaching focusing on small steps and experiences of success - providing schools with methods suitable for participation
3	Designing and implementing tailored activities	- conducting a needs assessment with *Photovoice*, along with interviews that can serve as input for designing activities - having coordinators organize *design-thinking* sessions with representatives of all stakeholders - providing an implementation budget as a start-up resource
4	Creating a network of local support	- giving experts from local organisations in different areas of expertise an active role in supporting school communities - coaching from experts aims towards building potential collaborations within the local network throughout the intervention, based on the needs of the community

The second strategy (creating a school culture of participation) is facilitated by the coordinators, with support from two experts in the field of health promotion and education. The experts coach the healthy-school coordinator in regularly addressing the topics of PA and dietary behaviour, in addition to informing members of the school community about how they can contribute and participate on this topic. Coaching focuses on starting with small actions, expanding their number and content according to the coordinators’ personal strengths, available opportunities and success experiences. Furthermore, schools are provided with information about existing participatory methods that could potentially stimulate community change [47].

The third strategy (designing and implementing tailored activities) is facilitated by coordinators, who organize goal-setting sessions with groups of representatives from the community. The *design-thinking* method [48] is used for this strategy, as it places the community in a central position and focuses on the joint creation of concrete actions that fit the specific context of the community [49]. Participants in these sessions generate ideas by brainstorming and making at least one idea as concrete as possible, including a specification of the necessary roles and resources. The session concludes with an action plan for implementing the activities. The healthy-school coordinator then further specifies the action plan, with assistance from the two experts, who contribute their expertise and knowledge of evidence-based intervention strategies. Each school community is allocated a budget for facilitating activities.

The fourth strategy (creating a local supportive network) is facilitated by the two experts, who work in local organizations in the fields of both health promotion and education. Each participating school community worked with these organizations with regard to the regular Dutch Healthy School Approach before the start of the FLASH intervention, and they will continue to do so after the intervention. During the intervention, these experts devote particular attention to working with the healthy-school coordinators to build a local network of organizations and partnerships, such as the municipality or sport clubs. These networks could potentially assist school communities in structurally embedding specific activities by providing resources and partnerships.

#### Output and outcome: continuous improvement processes

The FLASH intervention follows an action-oriented approach. With regard to outputs, we monitor processes of community-capacity building, the implementation of tailored activities and the influence of contextual factors on these processes in real-life settings. The observed results are used to achieve the continuous improvement of processes within capacity-building strategies. Building community capacity and implementing tailored activities are treated as two reciprocal processes that can influence each other. Successful activities can increase community capacity, and increased community capacity can help the school community to replace or adjust less successful activities [50]. As outcomes, the PA and dietary behaviour of students and their attitudes towards PA and nutrition are reported back to the community each year for purposes of adjusting and improving capacity-building strategies.

### Study population and recruitment

Eight pre-vocational schools are participating in the study, with four following the FLASH intervention and four following the regular Dutch Healthy School Approach. The number of intervention schools was guided by pragmatic reasons relating to inputs for building community capacity (e.g. staffing, equipment, available time), in order to achieve the desired intensive following of intervention schools to identify working strategies for capacity-building. Schools with different characteristics were included to ensure the possibility of comparing how different school communities deal with similar opportunities, barriers and conditions. Differences in school characteristics relate to size, rural/urban environment and types of pre-vocational educational offered. Control schools are matched on these characteristics to allow comparison between a community-based approach and the regular Healthy School Approach.

The FLASH intervention schools are located in the North-eastern region of the Netherlands. All intervention schools are part of the educational organization that was a co-developer of this intervention, and they were recruited by a staff member from this organization. The four pre-vocational schools that agreed to the following selection criteria were invited to participate: 1) commitment to intervention and evaluation for 4 years, 2) willingness to facilitate new health-promotion activities based on the Healthy School pillars on the themes of healthy PA and nutrition, 3) willingness to appoint a staff member of the school to coordinate the FLASH intervention. Intervention schools are compensated through the intervention inputs for capacity building (Fig. 1). Two schools are located in urban environments, and two are located in rural environments. One of the rural schools and one of the urban schools offer only pre-vocational education. The other two schools also offer higher types of secondary education which prepares students for higher professional education or university. The range of the total pre-vocational student population in each school is between 120 and 600 students.

The control schools are located outside of the North-eastern region of the Netherlands, thus preventing contamination of the results due to connections between these schools and the educational organization. Control schools were recruited by the research team through networking, and they were selected according to matching with characteristics of the intervention schools. Each control school receives €500 for participation if all measurements in the effect evaluation are completed.

#### Study population for process evaluation

The study population for the process evaluation is made up of the three stakeholder groups in the intervention schools: pre-vocational students, school personnel, and parents. Parents in school communities that also offer other levels of education must have at least one child enrolled at the pre-vocational level. In each intervention school, purposive sampling [51] is used to collect data on changes in community capacity. Key stakeholders are identified by the healthy-school coordinator. At least six to eight stakeholders are recruited in each community to ensure a complete overview of different perspectives [44]. The inclusion of key stakeholders will continue until data saturation has been achieved.

Participants and organizers of tailored health-promotion activities implemented in each school are questioned in order to evaluate the activities and the community involvement in them. To gain insight into the daily processes for building community capacity throughout the intervention, healthy-school coordinators and experts are asked to provide regular input through digital channels.

#### Study population for effect evaluation

The study population for the effect evaluation is made up of all pre-vocational students starting their second year in either an intervention or control school in 2016, 2017 or 2018. Second-year students were selected as the target group, as they are familiar with the school community and likely to remain in it for at least three more years until graduation. Participating students provide written consent by completing a preliminary question before starting the questionnaire. Students can only participate if their parents/guardians provide written consent as well. The school sends the parents of eligible students an information letter and consent form. Depending on the size of a school, between two and six pre-vocational classes of approximately 20 students are approached each year. Participating students will be included in the study until they leave school or until the study ends (March 2020). The population will be divided into three cohorts, based on the year in which students entered the study.

### Data collection and outcomes

#### Process evaluation

Outcomes relating to changes in community capacity, health-promotion activities and the monitoring of daily processes in capacity-building are based on the RE-AIM framework (see Fig. 1) [45]. Table 2 provides an overview of data-collection methods, frequency, population and outcomes for the elements Reach, Effectiveness, Adoption, Implementation and Maintenance.

**Table 2:** Outcome measures of process evaluation based on RE-AIM framework

Goal	Method, Population and Frequency	Reach	Effectiveness	Adoption	Implementation	Maintenance
Assessing changes in community capacity	Semi-structured interviews with stakeholders (*N* = 6–8, at start and end of intervention)	Subjective reach of FLASH as identified by interviewees	Comparing scores for community capacity composed at start and end of intervention	Number of people involved in implementing FLASH intervention	Evaluation of used strategies to increase community capacity	Evaluation of resources and plans to structurally embed a community based approach
Assessing changes on health promotion activities	Questionnaires for assessing activities on each pillar: education, policy, environment, monitoring (filled out by coordinator at start and end of intervention)		Changes in activities with regard to health education, policy, environment (social and physical), and monitoring. Assessed on number and content		Evaluation of who was involved in changes with regard to the pillars and how were changes achieved	
	Observational scans of physical environment: canteen scan (nutrition) and school yard scan (PA) (performed by trained researcher at start and end of intervention)		Changes in physical environment		Evaluation of who was involved in changes and how changes came to be	
	Questionnaire and/or focus group among initiators and target group of specific activities in a school community (third year)	Number of stakeholders participating in activities	Evaluation among target group to what extent aim of an activity is achieved	Number of people involved in implementing an activity	Evaluation with key stakeholders of each communities’ action plan vs what happened	Evaluation with leaders and key stakeholders if and how actions can be repeated and become structurally embedded
Monitoring of daily processes for capacity building	School coordinator journals (every 2 months)		Satisfaction in the role of coordinator		How the available hours were used and how expert roles were used	
	Document analysis (minutes of meetings, continuous during 3 years)				Experiences on barriers, opportunities and conditions for implementation	Identifying opportunities for structural embedding
	Interviews and questionnaires with key-stakeholders (third year)				Experiences on barriers, opportunities and conditions for implementation	Views on opportunities conditions necessary for continuation and adaption

##### Changes in community capacity

The primary outcome of this study is change in community capacity. The *community readiness to change* interview method [44] is used to assess this capacity. This semi-structured interview method is based on the four capacity-building strategies used in the FLASH intervention. It includes in-depth questions concerning: 1) leadership, initiators and roles in creating a healthy community; 2) school culture with attention to and participation in PA and dietary behaviour activities; 3) processes and conditions for designing and implementing activities and for raising awareness about these activities within the community; and 4) local networks and resources that could be used for structural implementation. Interview questions are translated into Dutch, discussed in the research team and pre-tested among the healthy-school coordinators. In each school community, key stakeholders (*N* = 6 to 8) are interviewed in their capacities as school personnel or parents.

The method includes an analysis and scoring system to generate a score for community capacity. This system has been validated [44] and previously used as an indicator of community capacity [50, 52]. The scoring system is used to assess the Effectiveness element of the RE-AIM framework by performing the interviews at the start and end of the FLASH intervention and by comparing the community-capacity scores. The resulting score is illustrated with additional inductive analysis to assess the Implementation element with regard to how each school community carried out the four capacity-building strategies within their specific contexts. Data are also assessed concerning a) the Reach element, with regard to the number of people familiar with FLASH; b) the Adoption element, with regard to the number of people actively involved in creating the healthy-school community and; c) the Maintenance element, with regard to plans and resources for structurally embedding the community-based approach.

##### Changes in tailored health-promotion activities

The activities of each school community are evaluated on how they fit within the pillars of the Dutch Healthy-School Approach: Education, Policy, Environment and Monitoring. We consider the number and content of activities within each pillar to assess the Effectiveness element of a community-based intervention for creating tailored activities. We assess the Implementation element by evaluating who was involved and how changes emerged. These elements are assessed using questionnaires that each healthy-school coordinator completes at the start and end of the intervention. To assess changes in the physical Environment pillar, a canteen scan [53] and a schoolyard scan [54] are conducted by trained researchers. Each specific activity is also evaluated by questionnaires or focus groups amongst the target group and stakeholders who had organized activities in order to determine the extent to which they achieved goals that had been set by the community.

##### Monitoring of processes with regard to capacity building

Daily processes during the FLASH intervention are monitored and evaluated every other month according to journals maintained by the healthy-school coordinators, as well as by document analysis of meetings held throughout the intervention and interviews with key stakeholders from local organizations (e.g. municipality, local public health service, educational organization). We assess the satisfaction of the healthy-school coordinators in their roles as community leaders, as well as how they utilized the inputs of available hours and support by experts. Document analysis is used to collect data on barriers, opportunities and conditions regarding the implementation and maintenance of a community-based approach. These data are supplemented with data gathered through interviews with key stakeholders from local organizations to assess opportunities for and barriers to implementation and maintenance.

#### Effect evaluation

The measures used for the effect evaluation are secondary outcomes. A questionnaire on self-reported health behaviours and anthropometric measurements are conducted annually amongst pre-vocational students in the intervention and control schools, in order to provide an initial indication of the effect of this intervention. Data are gathered in three cohorts between October and November of the years 2016, 2017, 2018 and 2019 (see Table 3). Outcomes are related to PA, dietary behaviour (including attitude towards PA and dietary behaviour), BMI and waist circumference.

**Table 3:** Effect evaluation measurements, by cohort

	Cohort 1 *(started the second year in Sept. 2016)*	Cohort 2 *(started the second year in Sept. 2017)*	Cohort 3 *(started the second year in Sept. 2018)*
School year 2016/17	Measurement 1	–	–
School year 2017/18	Measurement 2	Measurement 1	–
School year 2018/19	Measurement 3	Measurement 2	Measurement 1
School year 2019/20	–	Measurement 3	Measurement 2

##### Questionnaire

The questionnaire is administered digitally during school hours in a classroom setting. A researcher and a teacher are present at all times. Oral instructions are recorded and incorporated into the questionnaire as audio files, in order to ensure that all students receive the same instructions on how to complete the items. The questionnaire includes questions about demographic characteristics, PA and dietary behaviours, and attitudes towards health. It is based in part on a validated questionnaire assessing behaviours relating to energy balance in Dutch adolescents [55], and it was pre-tested amongst pre-vocational students of various ages.

With regard to demographic characteristics, questions address the gender, age, type of education (including level and year), living situation and country of birth of students, as well as of their mothers and fathers. In the Netherlands, prevocational educational is divided into four levels that differ in the ratio of practical vocational training and theoretical education. Living situation is divided into five categories: living with both parents, living primarily with the father, living primarily with the mother, living arrangements divided between father or mother, or living with another guardian.

With regard to PA and dietary behaviour, questions address physical activity, sedentary behaviour, sleep and dietary behaviour. Each theme is divided into several topics. For physical activity, we consider active transport to school, exercise during free time and total activity per week. For sedentary behaviour, we consider gaming, social-media use and watching TV/other screen time. For dietary behaviour, we consider healthy dietary behaviour (breakfast, fruit, vegetables, water) and unhealthy dietary behaviour (sugar-sweetened beverages, snacks, candy). With regard to attitudes towards health, questions address attitudes towards PA and dietary behaviour, the influence of the social and physical environment on PA and dietary behaviour, and attitudes towards education on PA and dietary behaviour. Additional file 1 provides an overview of each theme and topic, along with contributing questions and response categories.

##### Anthropometric measurements

Weight, height and waist circumference are measured by trained research assistants according to a protocol [56]. Students remain clothed, but are asked to remove large or irregular clothing items. Weight is measured to the nearest 0.1 kg (weighing scale 888, Seca, Germany). Height is measured to the nearest 0.1 cm (stadiometer 217, Seca, Germany). Waist circumference is measured to the nearest 0.1 cm using a measuring tape [57]. Weight and height are used to calculate BMI z-scores, based on Dutch reference values [56].

##### Sample size calculation

The sample size calculation was based on the outcome regarding a difference in mean absolute BMI z-score between the intervention and control group after 2 years of the intervention. Taking into account an Intraclass Correlation Coefficient within schools of 0.10, an alpha of 0.05, power of 0.80 and loss-to follow-up of 20%, we will include 200 students in the intervention schools and 200 students in the control schools to ensure that a mean difference in BMI-score of 0.35 (standard deviation of 1.2) between the intervention and control group will be statistically significant.

### Analyses

Interviews for assessing community capacity are analysed according to an anchored scoring system and using an inductive thematic approach. The anchored scoring system enables us to create a score to indicate the level of community capacity in each school community. Interviews are scored for a stage of readiness for community change, ranging from no awareness and denial/resistance to expansion and a high level of ownership [44]. Each of the four capacity-building strategies is scored on level of readiness. These scores can be combined into a total score representing the status of community capacity within each school. Total scores are compared at the start of the intervention and after 2 years. The inductive thematic approach uses open coding to assess themes regarding barriers to and opportunities and conditions for creating a healthy-school community.

Questionnaires assessing changes in health-promotion activities are analysed according to descriptive statistics. Open-ended questions and focus groups are analysed according to an inductive thematic approach. Observational scans of the physical environment are analysed according to the tool’s analytical system: a) the canteen scan classifies the food environment into levels reflecting the relative healthiness of the canteen and vending machines [53], and b) the schoolyard scan measures the availability of exercise options in number and type [54]. To assess the monitoring of processes for building community capacity, we analyse logbooks maintained by healthy-school coordinators using descriptive statistics. Open-ended questions, interviews and minutes from meetings are analysed according to an inductive thematic approach.

Coding for all qualitative data (using either the anchored scale or inductive thematic approach) is conducted independently by two researchers in a qualitative analytical program. Differences are discussed with a third researcher. All researchers maintain reflective diaries during the data-collection and coding phases, in order to evaluate their own subjective views when interpreting data. Interviews and focus groups are audio-recorded and transcribed. Quantitative data collected for the effect evaluation are analysed using multilevel analysis. We will assess the necessity of correcting for dependency between schools.

## Discussion

This paper describes the study protocol for monitoring and evaluating the FLASH intervention, which aims to build community capacity and empower school communities to design and implement tailored health-promotion activities. These activities are aimed specifically at encouraging healthy PA and dietary behaviours among pre-vocational students. The study focuses primarily on examining changes in community capacity and evaluating processes for designing and implementing activities developed in each school community, based on the Dutch Healthy School pillars. A secondary objective is to provide an indication of the effect of this community-based school health-promotion intervention on PA, dietary behaviour (including attitude towards PA and dietary behaviour) and overweight among pre-vocational students.

The main strengths of this study are that it uses mixed methods (i.e. triangulation) increasing the internal validity and an action-oriented approach to evaluate real-life situations within a school setting. We systematically gather information on *how* schools build their community capacity and how they design and implement activities, thereby identifying ‘best practices’, instead of merely tracking *what* is done [35]. This can help to increase the applicability of evidence-based interventions, which do not always yield results in real-life situations [21]. As a result of the action-oriented approach, each school is allowed to adapt the provided inputs for facilitating capacity-building to their specific context and school culture. This also entails that each school will follow different processes of building community capacity and will create and implement different health-promotion activities. These school-specific processes and activities will not be generalizable to other schools. However, the observational nature of this study does provide a substantial amount of information on processes of knowledge accumulation and how stakeholders arrive at specific actions [35, 58]. This type of information can subsequently be translated into working strategies that can be applied to a larger number of schools [58].

To our knowledge, no community-based approach focussing on building community capacity has been used to date in the field of health promotion in school settings in the Netherlands. Because the primary focus of this study is to develop working strategies for building community capacity, we included a relatively low number of intervention schools (*N* = 4) to enable us to follow processes in these schools intensively. To increase the likelihood of a broad understanding of strategies for building community capacity, we included intervention schools with different characteristics in terms of physical environment, culture and population. Although the quasi-experimental design based on this number of intervention schools and a similar number of control schools prevents us from drawing definite conclusions concerning causal relationships between the FLASH intervention and changes in health behaviour over time, it does provide an initial indication of the effectiveness of a community-based approach within a school setting.

It should be noted that, in this study, health behaviours are assessed through a self-reported questionnaire, which could result in socially desirable answers. This could also lead to selection bias [59], due to the lack of randomization at the participant level. To assess this bias, sociodemographic characteristics and results will be compared to regional data from the local public-health service, which periodically gathers measurements on BMI, PA and dietary behaviour among students in the first or second years of secondary school.

This study will provide insight into how school communities can design and implement sustainable, tailored health-promotion activities by building their community capacity. By focusing on real-life situations and using mixed methods to gain in-depth insight into processes relating to barriers, opportunities and conditions, the results could enable schools to adapt and adopt integral health-promotion interventions in daily practice that suit the needs of the communities. This may lead to more sustainable interventions and that could have a positive influence on adolescent health in terms of PA and dietary behaviours.

## Additional file

Additional file 1: Overview of themes, topics and response categories of self-reported questionnaire in the FLASH intervention. This file provides an overview of the questionnaire used in the evaluation study of the FLASH intervention with regard to PA and dietary behavior and determinants of these behaviors. (PDF 98 kb)

## Data Availability

Not applicable.

## Abbreviations

FLASH intervention: Fit Lifestyle at School and at Home intervention
PA: Physical Activity
