# Supplementary material for: Background and evaluation design of a community-based health-promoting school intervention: Fit Lifestyle at School and at Home (FLASH)
Source: BMC Public Health. 2019 Jun 20;19:784. doi: 10.1186/s12889-019-7088-3 (PMC6585041; doi:10.1186/s12889-019-7088-3)
Supplement: Supplementary file 1 — Overview of themes, topics and response categories of self-reported questionnaire in the FLASH intervention. This file provides an overview of the questionnaire used in the evaluation study of the FLASH intervention with regard to PA and dietary behavior and determinants of these behaviors. (PDF 98 kb) [file 12889_2019_7088_MOESM1_ESM.pdf]

Additional file 1 – overview of themes, topics, questions and response categories of the self-reported questionnaire among students in the FLASH intervention

HEALTH BEHAVIORS			
**THEME**	**Topic**	**Question**	**Response categories**
**PHYSICAL ACTIVITY**	- Active transport<br>- Exercise during free time<br>- PA a week	- On how many days: do you walk or bike to school/ do you exercise at a club/ do you exercise without a club?<br>- How long do you walk or bike to school?<br>- How many days do you exercise at a club/ without a club?<br>- How many days do you exercise at least one hour?	- (almost) never/ 1/2/3/4/5 day(s) a week<br>- I don't walk or bike/1-5/6-10/11-15/16-30/31-45/more than 45 minutes<br>- (almost) never/ 1/ 2-3/ 4-5/ (almost) every day a week<br>- (almost) never/1/2/3/4/5/6/7 days a week
**SEDENTARY BEHAVIOR**	- Gaming<br>- Social media<br>- Watching TV/screen time	- How many times do you play games/ use social media/ watch programs on TV or other screen?<br>- How long do you play games/ use social media/ watch programs on TV or other screen on an average week day?<br>- How long did you play games/ use social media/ watch programs on TV or other screen yesterday?	- (almost) never/ 1/ 2-3/ 4-5/ (almost) every day a week<br>- Not at all/0.5/1/2/3/4/5/6 or more hour(s) a day<br>- Not at all/0.5/1/2/3/4/5/6 or more hour(s) a day
**SLEEP BEHAVIOR**	Sleep behavior	- What time do you go to bed on a weekday?<br>- What time do you go to sleep on a weekday?<br>- What time do you week up on a weekday?	- 21.00 or earlier/ 21.30/ 22.00/ 22.30/ 23.00/ 23.30/ 00.00/ 00.30/ 01.00 or later<br>- 21.00 or earlier/ 21.30/ 22.00/ 22.30/ 23.00/ 23.30/ 00.00/ 00.30/ 01.00 or later<br>- 6.00 or earlier/ 6.15/ 6.30/ 6.45/ 7.00/ 7.15/ 7.30/ 7.45/ 8.00/ 8.15/ 8.30/ 8.45/ 9.00 or later
**DIETARY BEHAVIOR**	- Healthy dietary behavior (breakfast, fruit, vegetable, water)<br>- Unhealthy dietary behavior (sugar sweetened beverages, snacks, candy)	- How many days a week do you eat breakfast/ eat fruit/ eat vegetables/ drink water?<br>- How much water do you drink on an average weekday?<br>- How much water did you drink yesterday<br>- On how many days do you eat/drink regular soda/ fruit juices/ snacks/ candy?<br>- How much regular soda (small/large)/ fruit juices (small/large)/ snacks/candy do you eat/drink on an average weekday?<br>- How much regular soda (small/large)/ fruit juices (small/large)/ snacks/candy did you eat/drink yesterday?	- (almost) never/1/2/3/4/5/6/7 days a week<br>- 0/1/2/3/4/5/6/7 or more glasses<br>- 0/1/2/3/4/5/6/7 or more glasses<br>- (almost) never/1/2/3/4/5/6/7 days a week<br>- 0/1/2/3/4/5/6/7 or more glasses or small bottles/ cans or big bottles/ snacks/ candy<br>- 0/1/2/3/4/5/6/7 or more glasses or small bottles/ cans or big bottles/ snacks/ candy

DETERMINANT OF HEALTH BEHAVIOR: ATTITUDE		
**THEME**	**QUESTION**	**RESPONSE CATEGORY**
**TOWARDS HEALTH**	What do you think about regular soda/ energy drinks/ fruit juices/ eating snacks/ eating candy/ drinking water/ eating breakfast daily/ eating fruit/ exercising 1 hour a day/ gaming every day/ using social media every day/ watching programs every day/ waking or biking to school every day?	Very good/ good/ not bad or good/ bad/ very bad
**TOWARDS SOCIAL ENVIRONMENT (PARENTS)**<br>- HAVING RULES<br>- AVAILABILITY<br>- MODELING<br>- ENCOURAGEMENT	1.Do your parents have a rule about: eating breakfast/ fruit/ vegetables/ candy/ snacks/ drinking sodas or fruit juices/ energy drinks/ how long you can spend on a screen/ engaging in a sport/ walking or biking to school? (if yes, then a how many times a month do your parents keep you to this rule was prompted)<br>2.How many days a week are there sodas or fruit juices/ candy/ snacks/ fruit in the house?<br>3.How many times does your father/mother/other exercise/ use a screen at home/ eat candy/ eat snacks/ drink soda or fruit juice/ drink water?<br>4.How many times do your parents stimulate you to exercise/ use a screen les/ eat less snacks or candy/ drink less soda or fruit juice/ drink more water?	1.Yes/ No (less than once/ once/ multiple times a month/ multiple times a week)<br>2.Less than once/ once/ 2-3/ 4-5/ 6-7 days a week<br>3.Less than once/ once/ 2-3/ 4-5/ 6-7 days a week<br>4.Less than once/ once/ multiple times a month/ multiple times a week/ every day
**TOWARDS SOCIAL ENVIRONMENT (PEERS)**	To what extent do your friends exercise at a club/ exercise on the street/ walk or bike to school/ engage in more than 3 hours of screen time/ use social media/ eat snacks/ eat candy/ drink soda/ energy drinks/ fruit juices/ water/ bring fruit to school/ eat breakfast daily/ buy snacks in school canteen/ buy snacks in supermarket	Totally agree/ agree/ don't disagree or agree/ disagree/ totally disagree
**TOWARDS PHYSICAL ENVIRONMENT**	1.Our school canteen/vending machines has/have enough healthy products<br>2.There are enough places I can fill my water bottle/ be physically active<br>3.During break time I have enough time/space to eat my lunch<br>4.I regularly buy healthy products in the canteen/vending machines/supermarket<br>5.How often do you buy sodas, fruit juices or energy drinks/ snacks or candy at school/at the supermarket	1-4: Totally agree/ agree/ disagree/ totally disagree<br>5: (almost) never/ 1/ 2-3/ 4-5/ 6-7 days a week
**TOWARDS HEALTH EDUCATION**	1.I want to learn how to make healthy food/ make a healthy choice/ whether I eat healthy<br>2.I like our lessons at school about healthy food/ about Physical Education (PE)<br>3.Lessons at school made me eat more healthy/ made me exercise more in my free time/ gave me an idea of the type of sport I want to do/ learned me about healthy PA<br>4.I like more PE lessons/ I am active during PE lessons	1-4: Totally agree/ agree/ disagree/ totally disagree
